# La Charité

**Published:** 1833-03

**Authors:** 


### LA CHARITÉ.

### *Death from a single Leech-bite.*

A boy was attacked with symptoms for which it was deemed proper to apply a dozen leeches to the pit of his stomach, and, when they had dropped off, some burnt linen was put to the parts, and the patient was left for the remainder of the day. When he was visited, his bed was found full of blood, and the hemorrhage continued in spite of every attempt to arrest it. Three and twenty hours after the leeches were applied, he was carried to La Charité. The abdomen was covered with one enormous clot, and it was found that the bleeding proceeded from *one* leech-bite, situated a little above the navel. The blood was arterial. Nitrate of silver was in vain applied, and the actual cautery was had recourse to, but not until the boy was too far exhausted; the extremities were cold, pulse nearly gone, and the voice extinct. He died in two hours after his admission.

*Dissection.* Nothing remarkable was observed, except the absence of blood from the heart and all the tissues. M. Bricheteau calculated that the quantity of blood lost amounted, at least, to three pounds; and, for the purpose of ascertaining, as far as possible, the correctness of his opinion, he collected the blood that flowed from a leech-bite in the thigh, and which was suffered to flow for some hours. In ten minutes he obtained three drachms, which gives two ounces and two drachms for the hour; and hence the inference that more than three pounds was lost in the above case.—*Gazette des Hôpitaux.*

This case affords an additional proof to several others of a similar kind that have been reported in different journals, of the necessity of always looking at the punctures some time after the leeches have dropped off. No practitioner, we presume, would omit such a precaution when leeches have been applied to young children; but it should also be observed with respect to adults. Whether, in the above instance, the boy was so far exhausted, when the bleeding was first discovered, as to preclude the possibility of recovery, we cannot determine; but it appears to us that time was lost in the application of "styptics," when other means might have been instantly and effectually applied. The bleeding from a leech-bite may always be arrested by the pressure of a finger on the spot, and it rarely happens that it is necessary to continue the pressure longer than a few minutes. Even if hemorrhage be going on from several bites, the same mode of arresting it may be adopted; for the attendant may ensure effectual pressure by placing a finger over each bite. We have often stopped the bleeding from leech-bites in this manner, when children have been reduced to a very alarming state, and when various other means to arrest it had been tried in vain. In general, also, a very minute piece of sponge, inserted into the bite, and retained by a graduated compress, will at once stop the hemorrhage. The subject may appear trifling, and any suggestions in reference to it quite uncalled for; but we have known more than one instance in which the life of a child has been lost by long-continued hemorrhage from leech-bites, and we are confident that such accidents can never happen unless the practitioner is culpable, by his carelessness in not inspecting the part to which the leeches have been applied, or unless he loses time in applying ineffectual remedies, when either of those we have mentioned will stop the bleeding to a certainty.

### *Remarkable Case of Fatal Narcotism, produced by twelve drops of Laudanum introduced into the Rectum.*

The following case is perfectly without parallel, and is fraught with practical and medico-legal inferences of the highest importance. We derive the details from the *Gazette des Hôpitaux*:

"A man named Miolet, æt. forty-five, of sufficiently good constitution, was admitted to la Charité for a stricture of the rectum, and lay at No. 8, in the Salle St. Michael. On Tuesday, the 22d January, cauterization, according to the process of M. Costala, was employed in order to destroy the stricture. The operation gave great pain, but induced no other serious symptom. On the 24th, in order to moderate his dreadful sufferings, M. Rayer prescribed an injection of a decoction of marsh-mallows, and twelve drops of the laudanum of Sydenham. This decoction was given at six in the evening, and for some time the patient made no complaint, and during the evening he walked about the ward with the other patients. About nine P.M. he went to bed, and was scarcely laid down when he began to groan a little, and soon after fell asleep. This state lasted until two hours after midnight, at which time he was unable to answer the questions of the nurse. His intellectual faculties gradually grew weaker and weaker, his powers of sensation diminished, and he fell into a profound coma, from which, however, he appeared occasionally to emerge. In the morning, at M. Rayer's visit, he was found in the following state:

He lay on his back, plunged in a state of extreme prostration, his limbs absolutely immoveable, and in perfect collapse. The skin was covered with an abundant sweat. The eyelids fell, and the pupils were extremely contracted. The respiration was slow, and less than usually extensive, gradually becoming more embarrassed and sobbing. The pulse was very full, amounting to 110 per minute. What was rather rare was, that the urine was secreted in an unusually great quantity, so that his sheets were as wet as if dipped in water. The entire loss of the intellectual faculties, and of motion and perception, complete the description of this state. He was bled immediately to two cupsful, but immediately after the venesection his pulse still rose, and beat 150 in a minute. Water acidulated with vinegar was introduced almost mechanically into his stomach, and sinapisms were applied to his feet. But all efforts were useless, and he died at about eleven A.M.

The examination of the body was performed on the 26th, at ten A.M. The superficial cerebral veins, especially of the superior region of the brain, were gorged with thick black blood. The cerebral substance was a little moister than natural, but of natural consistence. There was no serosity in the lateral ventricles. The lateral sinuses were gorged with a thick black blood. Nothing particular on the superior longitudinal sinus, or in the spinal cord. The rectum was cancerous to the extent of two or three inches.

Encephaloid matter was observed on the surface of the mucous membrane, and scirrhous structure below it. The rest of the intestinal canal was slightly rosy. The mucous membrane of the stomach, however, was of a reddish brown aspect, and at the extremity of the upper surface of the right kidney there was a little cyst containing a serous and whitish fluid. The liver, especially on its upper surface, presented five or six cancerous patches of small extent. In the lungs there was seen nothing but slight congestion.

These facts permit no doubt as to the occurrence of narcotic poisoning. This is established, in the first place, by the character of the symptoms; secondly, by the presence of the pathological characters always attendant on this mode of death; and, thirdly, by the absence of any other pathological cause adequate to account for death under the circumstances before us. But how can we explain this event by the introduction of twelve drops of laudanum into the rectum? The evidence of numerous facts scarcely warrants us in coinciding in the opinion of M. Dupuytren, that opium acts in smaller doses in the rectum than in the stomach. This excellent surgeon generally administers it thus in nervous delirium. He believes that the opium is absorbed unchanged from the rectum, while it is partially digested in the stomach. However this be, we cannot but agree with the writer in our foreign contemporary, that the publication of this curious event is calculated to be of much use to medical practitioners, and we think with him that it is all but superfluous to add, that M. Rayer is free from the least blame on this unfortunate occasion.—*Lancet.*

